# High-Entropy and Component Stoichiometry Tuning Strategies Boost the Sodium-Ion Storage Performance of Cobalt-Free Prussian Blue Analogues Cathode Materials

**DOI:** 10.3390/molecules29194559

**Published:** 2024-09-25

**Authors:** Yuan-Ting Lin, Bai-Tong Niu, Zi-Han Wang, Yu-Xi Li, Yun-Peng Xu, Shi-Wei Liu, Yan-Xin Chen, Xiu-Mei Lin

**Affiliations:** 1 College of Chemistry, Chemical Engineering and Environment, Minnan Normal University, Zhangzhou 363000, China; 2 State Key Laboratory of Physical Chemistry of Solid Surfaces, College of Chemistry and Chemical Engineering, iChEM, Xiamen University, Xiamen 361005, China; 3 College of Energy, Xiamen University, Xiamen 361005, China; 4 Department of Physic, Xiamen University, Xiamen 361005, China; 5 State Key Laboratory of Structural Chemistry, Fujian Institute of Research on the Structure of Matter, Chinese Academy of Sciences, Fuzhou 350002, China; yanxinchen@fjirsm.ac.cn

**Keywords:** high-entropy, component stoichiometry tuning, prussian blue analogues (PBAS), cobalt-free cathode materials, sodium-ion batteries

## Abstract

Prussian blue analogs (PBAs) are appealing cathode materials for sodium-ion batteries because of their low material cost, facile synthesis methods, rigid open framework, and high theoretical capacity. However, the poor electrical conductivity, unavoidable presence of [Fe(CN)_6_] vacancies and crystalline water within the framework, and phase transition during charge–discharge result in inferior electrochemical performance, particularly in terms of rate capability and cycling stability. Here, cobalt-free PBAs are synthesized using a facile and economic co-precipitation method at room temperature, and their sodium-ion storage performance is boosted due to the reduced crystalline water content and improved electrical conductivity via the high-entropy and component stoichiometry tuning strategies, leading to enhanced initial Coulombic efficiency (ICE), specific capacity, cycling stability, and rate capability. The optimized HE-HCF of Fe_0.60_Mn_0.10_-hexacyanoferrate (referred to as Fe_0.60_Mn_0.10_-HCF), with the chemical formula Na_1.156_Fe_0.599_Mn_0.095_Ni_0.092_Cu_0.109_Zn_0.105_ [Fe(CN)_6_]_0.724_·3.11H_2_O, displays the most appealing electrochemical performance of an ICE of 100%, a specific capacity of around 115 and 90 mAh·g^−1^ at 0.1 and 1.0 A·g^−1^, with 66.7% capacity retention observed after 1000 cycles and around 61.4% capacity retention with a 40-fold increase in specific current. We expect that our findings could provide reference strategies for the design of SIB cathode materials with superior electrochemical performance.

## 1. Introduction

Energy storage devices are of great importance in the utilization and storage of renewable clean energy, including solar, wind, and hydro energy. Among various energy storage devices, lithium-ion batteries (LIBs) have dominated the electronic products and electric vehicle markets in the past three decades and achieved great success due to their mature fabrication technique, high energy density, and long cycling life. Nevertheless, the application of LIBs in large-scale energy storage devices is still confronted with significant challenges, especially the most important but high-cost and limited lithium and cobalt resources. Therefore, sodium-ion batteries (SIBs) couple a sodium metal anode with a cobalt-free cathode, showing appealing potential in cost and resources in large-scale energy storage devices [1,2]. Compared with lithium resources, the abundant and evenly distributed sodium resources lead to a lower price of SIBs than LIBs. In the meantime, the selection of an appropriate cathode material plays a pivotal role in the overall performance of a battery as it accounts for approximately one-third of the total battery cost and exerts a dominant influence on the output voltage, specific capacity, cycling life, etc. of a battery. Currently, the main SIB cathode materials include sodium-based transition metal oxides [3,4,5], phosphate-based polyanion [6,7,8], Prussian blue and its analogs (PB and PBAs) [9,10], and organics [11,12]. Among various SIB cathode materials, PBAs have attracted broad attention due to their facile, economic, and scale-up synthesis methods, rigid open framework, and high theoretical capacity [13]. However, the poor electrical conductivity, unavoidable presence of [Fe(CN)_6_] vacancies and crystalline water within the framework, and phase transition during charge–discharge of the material result in inferior electrochemical performance [14,15]. Traditional modification strategies of morphology construction, elemental doping, and surface coating have been applied to address these problems. However, they remain distant from the objective of developing high-performance SIB cathode materials.

Fortunately, the recent emergence of high-entropy (HE) materials, which are capable of maintaining a stable solid-state phase for use in energy-related applications, offers researchers unprecedented flexibility and variability in terms of materials composition and electronic structure. This has the potential to accelerate the development of new battery materials [16,17,18,19]. High-entropy battery materials (HEBMs) typically comprise a single phase comprising a substantial range of elements, with the interactions between these elements playing a significant role in enhancing battery performance. In comparison to conventional doping, which primarily involves the introduction of one or two elements, high-entropy approaches offer greater flexibility in structural design, facilitating the formation of a broader range of crystal and electronic structures [20,21]. Furthermore, the disruption of the crystal structure at the short-range level, which is a consequence of the presence of different elements, has two beneficial effects. Firstly, it increases the ability of the structure to evolve structurally during electrochemical processes. Secondly, it simultaneously induces defects that are beneficial for the migration of electrons and ions. As a result, the specific capacity, cycling stability, and rate capability of batteries are all improved [16,17,18,19,20].

The HEBM design process encompasses two principal aspects: the selection of essential elements and the determination of component stoichiometry. Elemental selection involves the selection of elements with different functions such as electrochemically active ions contributing to the specific capacity or electrochemically inactive ions stabilizing the host structure throughout the Li^+^/Na^+^ de/intercalation process. For PBAs cathode materials, the function of involving elements such as Fe^2+^, Mn^2+^, Ni^2+^, Co^3+^, Cu^2+^, Cr^3+^, Mn^4+^, Sn^4+^, Sb^5+^, Zr^4+^, Li^+^, Na^+^, Mg^2+^, Zn^2+^, etc. has been intensively studied [22]. Research indicates that Fe and Mn atoms in HE-HCFs can improve the capacity of the material, while Ni, Cu, and Zn can enhance the stability [23,24]. However, the influence of component stoichiometry of HB materials on their sodium-ion storage performance has seldom been investigated based on our literature research. In addition to functionality, the price and environmental benefits are important factors to consider in the electrode materials market. Currently, a large portion of HE-HCFs SIB cathode materials contain Co [16,17,18,19], which is rare, toxic, and costly. Low-Co or even Co-free materials, including elements such as Mn, Fe, Ni, Cu, Zn, etc., therefore, show appealing potential in the future battery materials market.

**Scheme 1:**
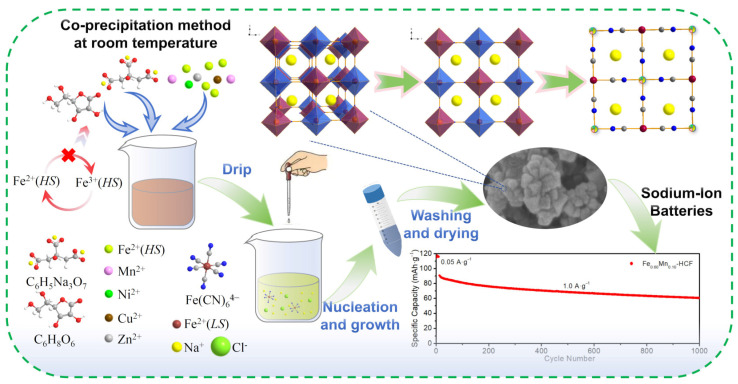
The schematic illustration depicts the synthesis procedure of Fe_x_Mn_0.70−x_-HCF materials via the co-precipitation method at room temperature and the crystal structure, SEM image, and sodium-ion storage performance of the Na_1.156_Fe_0.599_Mn_0.095_Ni_0.092_Cu_0.109_Zn_0.105_ [Fe(CN)_6_]_0.724_·3.11H_2_O (referred to as Fe_0.60_Mn_0.10_-HCF).

In this work, as illustrated in Scheme 1, cobalt-free single-metal PB of iron-based-hexacyanoferrates (referred to as Fe-HCF) and manganese-based-hexacyanoferrates (referred to as Mn-HCF) and HE-HCFs of Fe_x_Mn_0.70−x_Ni_0.10_Cu_0.10_Zn_0.10_-HCF (HE-HCFs) with a different component stoichiometry of Fe and Mn (referred to as Fe_0.60_Mn_0.10_-HCF, Fe_0.50_Mn_0.20_-HCF, Fe_0.35_Mn_0.35_-HCF, Fe_0.20_Mn_0.50_-HCF, and Fe_0.10_Mn_0.60_-HCF) cathode materials are synthesized using a facile and economic co-precipitation method at room temperature. Battery performance testing results of these materials show that the sodium-ion storage performance of the PBAs is boosted with the HE strategy, which is then further boosted by tuning the component stoichiometry of Fe and Mn. The optimized HE-HCF of Fe_0.60_Mn_0.10_-HCF having the chemical formula Na_1.156_Fe_0.599_Mn_0.095_Ni_0.092_Cu_0.109_Zn_0.105_ [Fe(CN)_6_]_0.724_·3.11H_2_O cathode material displays the most outstanding sodium-ion storage performance among all the synthesized PB and PBA cathode materials due to the reduced crystalline water content and improved electrical conductivity and kinetic demonstrated using electrochemical impedance spectroscopy (EIS) and galvanostatic intermittent titration technique (GITT) analysis. We expect that our findings could provide reference strategies for the design of SIB cathode materials with superior electrochemical performance.

## 2. Results and Discussion

### 2.1. Physical and Chemical Characterizations

The composition of the cobalt-free Fe-HCF, Mn-HCF, and HE-HCFs was determined using the inductively coupled plasma-optical emission spectroscopy (ICP-OES, Table S1), elemental analysis (EA, Table S2), and thermogravimetric analysis (TGA, Figure S1). The chemical formulas and corresponding abbreviated forms of all the synthesized PBAs are provided in Table 1. As illustrated in Figure S1, when the PB and PBAs were heated to 260 °C, the weight losses of Fe-HCF, Fe_0.60_Mn_0.10_-HCF, Fe_0.50_Mn_0.20_-HCF, Fe_0.35_Mn_0.35_-HCF, Fe_0.20_Mn_0.50_-HCF, Fe_0.10_Mn_0.60_-HCF, and Mn-HCF materials are 20.5%, 19.1%, 19.0%, 19.1%, 20.2%, 20.2%, and 16.8%, respectively. The Mn-HCF has a lower water content than the Fe-HCF and HE-HCFs. Nevertheless, the Fe-based HE-HCFs have lower water contents than single-metal Fe-HCF, which indicates that the high-entropy strategy effectively reduces the water content of PBAs. The crystal water content in PB or PBA materials has a significant effect on their unit cell parameters and structure stability. In PB or PBAs, water molecules can be filled in the voids of the crystal when they are introduced, which increases the unit cell parameters such as a, b, and c axis length and causes the expansion of the crystal. Moreover, the change in water content affects the structural stability of the crystal. Excessive water molecules may lead to deformation or destruction of the crystal structure and affect the stability of the cell parameters. Last but not least, the change in water content causes a thermal expansion effect. When the water molecules in the crystal exist in the form of crystal water, they are released during the heating process (drying of materials or electrode plates process), resulting in shrinkage of the cell parameters and even phase transition, which influences the structure stability and battery performance of the electrode materials [20,21,22,23,24]. The reduction of the water content in PBAs using a high-entropy strategy is, therefore, beneficial for the improvement of the electrochemical performance of the electrode materials.

**Figure 1:**
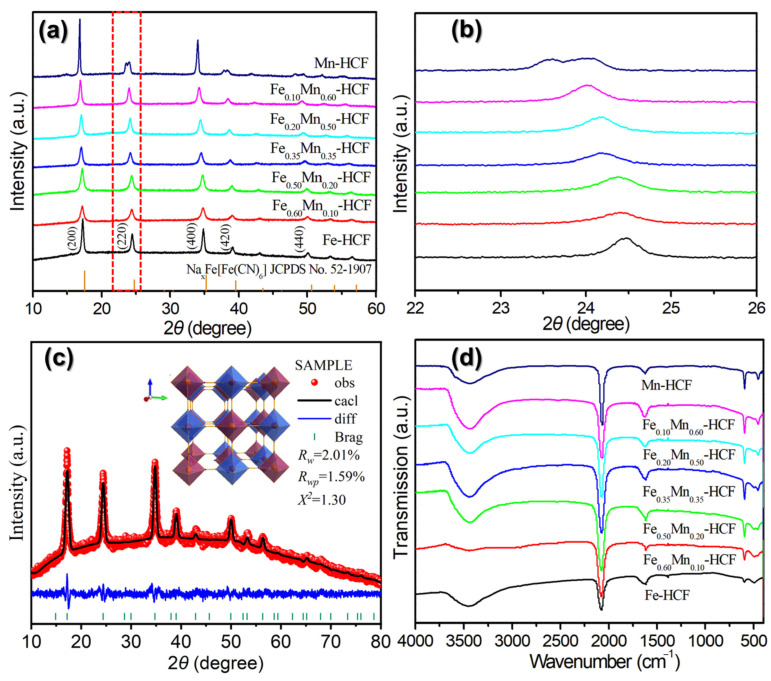
(**a**) XRD patterns and (**b**) The corresponding enlarged view at around 24° of the Fe-HCF, HE-HCFs (Fe_0.60_Mn_0.10_-HCF, Fe_0.50_Mn_0.20_-HCF, Fe_0.35_Mn_0.35_-HCF, Fe_0.20_Mn_0.50_-HCF, Fe_0.10_Mn_0.60_-HCF), and Mn-HCF materials. (**c**) The XRD corresponds to the Rietveld refinement profile, inserted is the schematic illustration depicts the crystal structure of Fe_0.60_Mn_0.10_-HCF material. (**d**) FT-IR spectra of the Fe-HCF, HE-HCFs, and Mn-HCF materials.

XRD spectra of Fe-HCF, HE-HCFs, and Mn-HCF are shown in Figure 1a. All these materials show main peaks at 2θ (in degree) values of around 17.1°, 24.2°, 34.5°, 38.8°, 49.7°, 53.1°, and 55.6°, corresponding to (2 0 0), (2 2 0), (4 0 0), (4 2 0), (4 4 0), (6 0 0), and (6 2 0) crystal planes of Na_x_Fe [Fe(CN)_6_] (JCPDS No. 52-1907) [25], which indicate t a single-phase cubic system with a space group of Fm-3m. Figure 1b is the corresponding enlarged view at around 24° of Figure 1a. As can be observed in Figure 1a,b, the peak positions of all the seven main XRD peaks gradually shift to smaller 2θ (in degree) values with the increase of the component stoichiometry of Mn to Fe of the PBAs (from bottom to top in Figure 1a,b), indicating the increase of the unit cell parameters and the interplanar spacing due the incorporation of Mn ion having larger ionic radius than that of Fe ion into the host Fe-HCF material. The Mn-HCF exhibits a similar characteristic XRD pattern to other synthesized PBAs except that the (220) peak at around 24° them splits into two peaks, suggesting a probable phase transition from cubic to rhombohedral phase (PDF card 04-022-3445) with space group R$\bar{3}$ [26]. The aforementioned different water contents of Fe-HCF, HE-HCFs, and Mn-HCF may be attributed to the different crystal types between them. XRD corresponds to the Rietveld refinement profile, and a schematic illustration of the crystal structure of Fe_0.60_Mn_0.10_-HCF material is shown in Figure 1c, whose lattice parameter is determined to be a = 10.3190(7) Å (Table S3), which increases slightly compared to that of the conventional Fe-HCF of a = 10.1990 Å. The lattice parameter is further increased to a = 10.3501(6) Å (Figure S2 and Table S4) with the further increase of the component stoichiometry of Mn to Fe in Fe_0.50_Mn_0.20_-HCF material. The structure of these materials was also characterized using Fourier transform infrared spectroscopy (FT-IR), shown in Figure 1d. Peaks at around 490 and 595 cm^−1^ correspond to the tensile vibration of M (Fe(HS), Mn, Ni, Cu, Zn)–N≡C and Fe–C≡N, respectively [27]. The strong peak at around 2074 cm^−1^ is associated with the tensile vibration of C≡N [28]. Peaks at around 1620 and 3445 cm^−1^ are associated with the H–O–H bending vibration and O–H stretching vibration of water molecules in the PBAs crystal structure, respectively [29,30,31,32]. 

**Figure 2:**
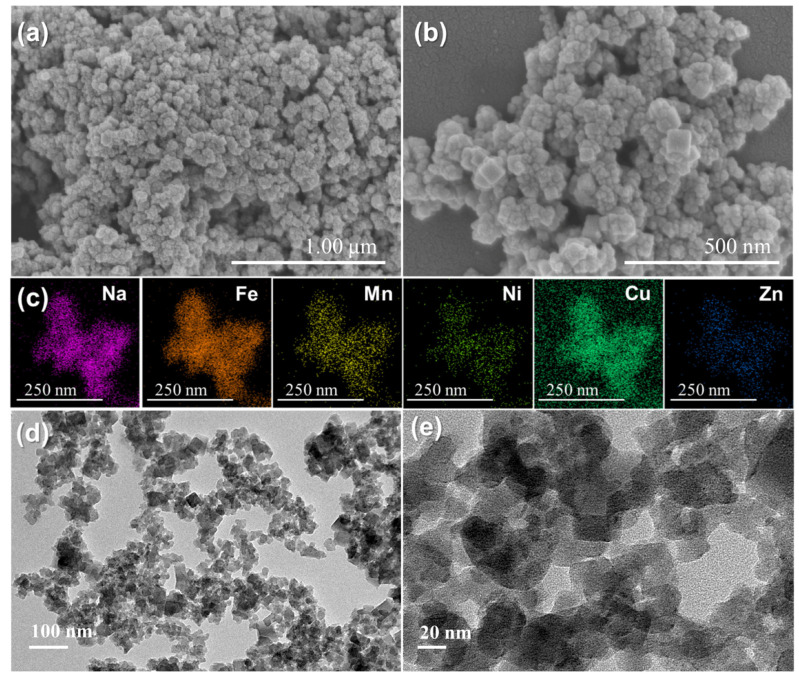
(**a**,**b**) SEM images. (**c**) Corresponding EDS elemental maps and (**d**,**e**) TEM images of the Fe_0.60_Mn_0.10_-HCF material.

Scanning electron microscope (SEM) images of Fe_0.60_Mn_0.10_-HCF with different magnifications are shown in Figure 2a,b, in which irregular particles with a size of around 50 nm aggregate together. SEM images of other PB and PBA materials are shown in Figure S3, showing similar aggregation states to Fe_0.60_Mn_0.10_-HCF. Interestingly, Fe-HCF has the smallest particle size of around 20 nm, the HE-HCFs have a medium particle size of around 50 nm, and Mn-HCF has the largest particle size of around 150–200 nm. The variation in particle size of Fe-HCF, HE-HCFs, and Mn-HCF could be attributed to the different stability constants of complexes formed between the different metal ions (Fe^2+^, Ni^2+^, Cu^2+^, Zn^2+^, Mn^2+^) with the sodium citrate complexing agent, which leads to different nucleation and growth rates and growth directions of PBAs [18]. Energy dispersive spectrometer (EDS) mapping images in Figure 2c show the evenly distributed elements of Na, Fe, Mn, Ni, Cu, and Zn in the Fe_0.60_Mn_0.10_-HCF material. Transmission electron microscope (TEM) images of Fe_0.60_Mn_0.10_-HCF with different magnifications are shown in Figure 2d,e, which further confirm that the material is constituted of irregular particles with an approximate diameter of 50 nm aggregated together and is consistent with the SEM results.

**Figure 3:**
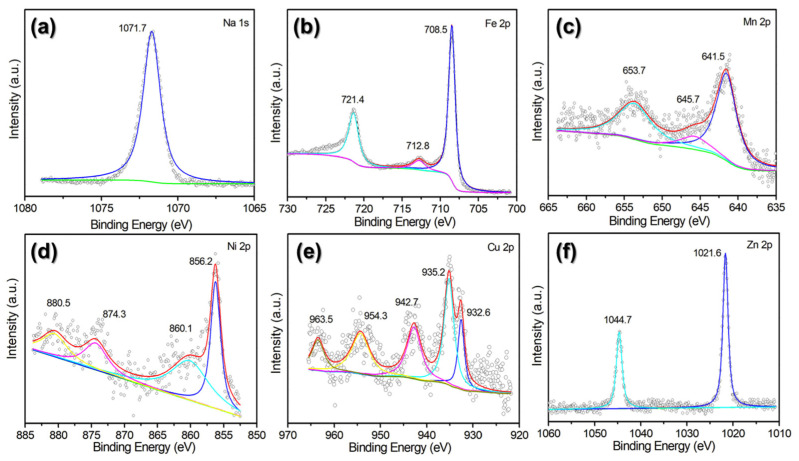
XPS detail spectra of the (**a**) Na 1s, (**b**) Fe 2p, (**c**) Mn 2p, (**d**) Ni 2p, (**e**) Cu 2p, and (**f**) Zn 2p regions for the Fe_0.60_Mn_0.10_-HCF material.

X-ray photoelectron spectroscopy (XPS) was employed to characterize the surface composition of Fe_0.60_Mn_0.10_-HCF material. The measurement confirms the existence of different elements, including Na, Fe, Mn, Ni, Cu, and Zn (Figure 3 and Figure S4). Figure 3a is the detailed spectrum of Na 1s, which shows a single peak with a binding energy of 1072.4 eV [33]. Figure 3b is the detailed spectrum of Fe 2p, and characteristic Fe 2p peaks appear at 708.5 eV (Fe 2p_3/2_) and 721.4 eV (Fe 2p_1/2_), with a spin-energy separation of 12.9 eV, indicating a 2+ oxidation state [34]. Additionally, a weak peak at 712.8 eV appears, which can be attributed to the 3+ oxidation state, indicating a small amount of Fe^3+^ on the surface of the material [35,36]. The proportion of Fe^3+^ is approximately 15.1% of the total Fe content. The peaks observed at 641.5 and 653.7 eV in the Mn 2p spectrum presented in Figure 3c can be attributed to the Mn 2p_3/2_ and Mn 2p_1/2_, respectively, while the peaks at 646.7 eV can be attributed to the shake-up satellites peaks (“sat”), indicating that Mn is in the 2+ oxidation state [37,38]. Similarly, Figure 3d shows the Ni 2p spectrum with peaks at 856.2 and 874.3 eV attributed to Ni 2p_3/2_ and Ni 2p_1/2_ of Ni^2+^, respectively, and two broad peaks at 860.1 and 880.5 eV as the shake-up satellites peaks [39,40]. The peaks observed at 935.2 and 954.3 eV in the Cu 2p spectrum presented in Figure 3e can be attributed to the Cu 2p_3/2_ and Cu 2p_1/2_, respectively, while the peaks at 942.7 and 963.5 eV can be attributed to the shake-up satellites peaks. Additionally, the peaks at 932.6 eV can be attributed to Cu 2p_3/2_ of Cu^+^, indicating that Cu in the material exists in the form of +1 and +2 oxidation states [41,42]. The proportion of Cu^+^ is approximately 14.0% of the total Cu content. Peaks at 1021.6 and 1044.7 eV in Figure 3f can be attributed to Zn 2p_3/2_ and Zn 2p_1/2_, which proves that the Zn element exists in the 2+ oxidation state [43,44]. Therefore, the average oxidation state of carbon-coordinated Fe(LS) and nitrogen-coordinated M (Fe(HS), Mn, Ni, Cu, Zn) cations are about 2+, which is consistent with the PB structure with linear chains of Fe^2+^–C≡N–M^2+^−N≡C–Fe^2+^. 

### 2.2. Sodium-Ion Storage Performance of the Synthesized PB and PBAs 

**Figure 4:**
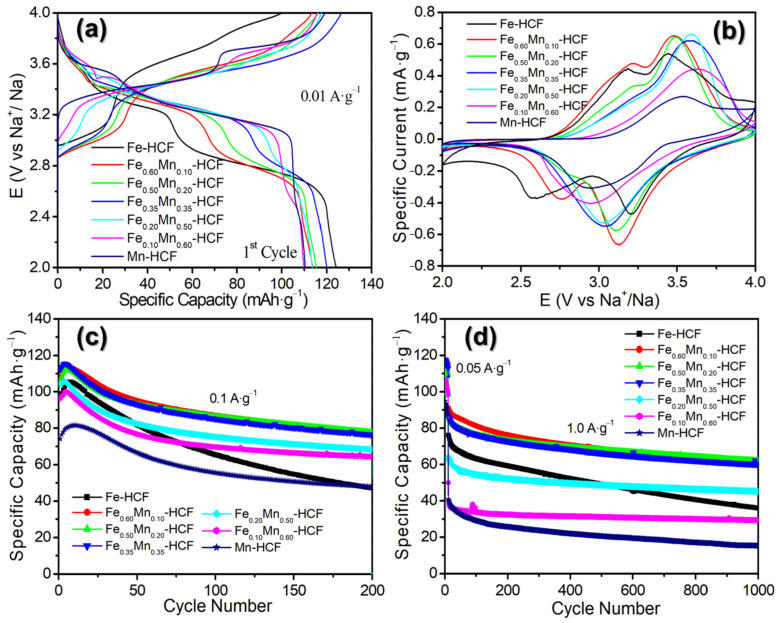
(**a**) Initial galvanostatic discharge–charge profiles at 0.01 A·g^−1^ in a Na half-cell (2.0−4.0 V), (**b**) CVs at 1.0 mV·s^−1^, and (**c**,**d**) Cycling stability at a specific current of 0.1 A·g^−1^ and 1.0 A·g^−1^ in a Na half-cell (2.0−4.0 V) of Fe-HCF, HE-HCFs, and Mn-HCF.

Figure 4a shows the galvanostatic discharge–charge profiles of the synthesized Fe-HCF, HE-HCFs, and Mn-HCF in a Na-half-cell at a specific current of 0.01 A·g^−1^. The discharge profile of Fe-HCF exhibits two plateaus at around 3.26 V and 2.82 V, respectively. These plateaus correspond to the oxidation of Fe with low spin states and high spin states. The discharge profile of Mn-HCF exhibits a plateau at around 3.20 V, corresponding to the oxidation of Mn [45]. For the HE-HCFs, with the increase of the stoichiometry of Mn to Fe from 1:6 to 6:1, the features of their corresponding discharge profiles transit from “Fe-HCF similar” to “Mn-HCF similar”. It is worth noting that the Fe_0.10_Mn_0.60_-HCF material has an obvious peak at about 3.45 V, which may be related to the serious phase transition of the material during the insertion/deintercalation of sodium ions. Their CV curves of HE-HCFs in Figure 4b show a similar transition phenomenon to the discharge profiles. The first discharge/charge capacities of Fe-HCF, Fe_0.60_Mn_0.10_-HCF, Fe_0.50_Mn_0.20_-HCF, Fe_0.35_Mn_0.35_-HCF, Fe_0.20_Mn_0.50_-HCF, Fe_0.10_Mn_0.60_-HCF, and Mn-HCF are 124/100, 114/113, 115/116, 120/127, 114/118, 109/116, and 110/119 mAh·g^−1^, respectively. They correspond to the initial Coulombic efficiency (ICE) of 124%, 100%, 99%, 94%, 97%, 94%, and 92%, respectively. Interestingly, Fe-HCF exhibits an ICE of 124%. The phenomenon of over 100% ICE can be attributed to the sodium-ion supplements from the sodium metal anode in half-cells, raised by the sodium-poor phase [46]. The ideal ICE for a cathode material should be close to or slightly lower than 100%, which is extremely important in full cells as the Na^+^ only comes from the cathode side. HE-HCFs approach 100% ICE more closely than single-metal Fe-HCF and Mn-HCF, and the Fe_0.60_Mn_0.10_-HCF presents the optimal ICE of 100%. Namely, the high-entropy and component stoichiometry tuning strategies synergistically improve the ICE of the PBAs SIB cathode materials. 

Figure 4c,d shows the cycling stability of Fe-HCF, HE-HCFs, and Mn-HCF at a specific current of 0.1 A·g^−1^ and 1.0 A·g^−1^, respectively, in a Na half-cell. At 0.1 A·g^−1^, after 200 cycles, the specific discharge capacities of Fe-HCF, Fe_0.60_Mn_0.10_-HCF, Fe_0.50_Mn_0.20_-HCF, Fe_0.35_Mn_0.35_-HCF, Fe_0.20_Mn_0.50_-HCF, Fe_0.10_Mn_0.60_-HCF, and Mn-HCF are 47.1, 77.5, 77.8, 76.3, 68.2, 64.3, and 47.9 mAh·g^−1^, respectively. Their corresponding specific capacity retentions are 48.9%, 72.8%, 74.0%, 68.1%, 65.4%, 66.8%, and 64.8%, respectively. Similarly, at 1.0 A·g^−1^, after 1000 cycles, the specific discharge capacities of Fe-HCF, Fe_0.60_Mn_0.10_-HCF, Fe_0.50_Mn_0.20_-HCF, Fe_0.35_Mn_0.35_-HCF, Fe_0.20_Mn_0.50_-HCF, Fe_0.10_Mn_0.60_-HCF, and Mn-HCF are 35.9, 60.6, 62.1, 60.0, 45.2, 29.4, and 15.0 mAh·g^−1^, respectively. Their corresponding specific capacity retentions are 47.4%, 66.7%, 72.5%, 70.3%, 70.6%, 58.8%, and 36.8%, respectively. Namely, HE-HCFs exhibit superior specific capacity and enhanced cycling stability compared to single-metal Fe-HCF and Mn-HCF at both low (0.1 A·g^−1^) and high (1.0 A·g^−1^) specific currents. Moreover, the Fe_0.60_Mn_0.10_-HCF and Fe_0.50_Mn_0.20_-HCF show the optimal specific capacity and cycling stability. Namely, the high-entropy and component stoichiometry tuning strategies synergistically improve the specific capacity and cycling stability of the PBAs SIB cathode materials. Variation in the average working voltage of the HE-HCFs materials during the electrochemical cycling process is shown in Figure S5. It can be observed that increasing the Fe(HS)/Mn ratio in the HE-HCFs materials improves their corresponding average operating voltage during the electrochemical cycling process.

**Figure 5:**
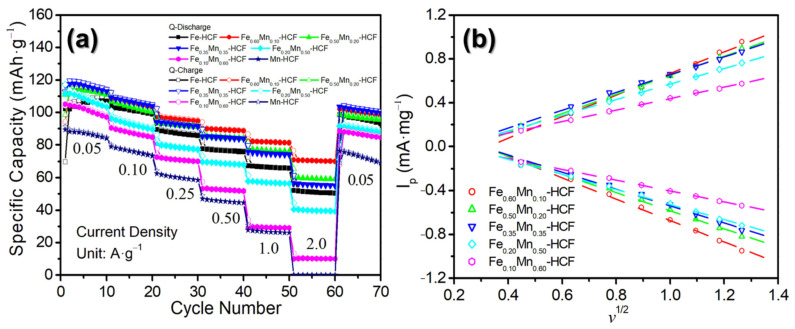
(**a**) Rate capabilities of the Fe-HCF, HE-HCFs, and Mn-HCF in a Na half-cell. (**b**) The linear fitting Randles–Sevcik curves of HE-HCFs by plotting the specific peak current as a function of the square root of the sweep rate for determining the apparent sodium-ion diffusion coefficient.

**Figure 6:**
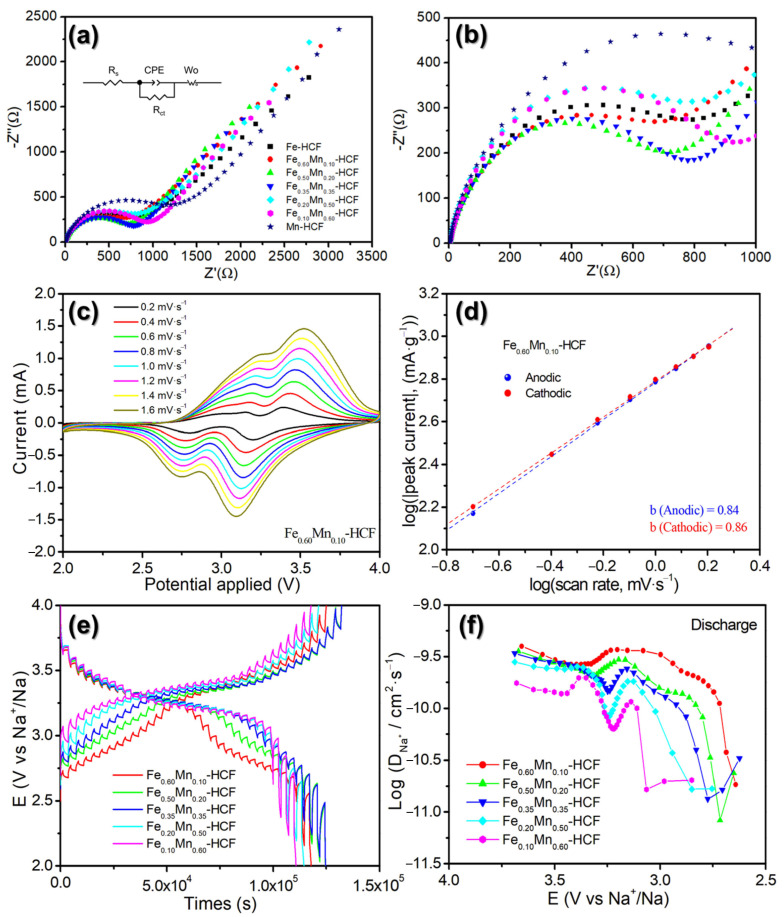
(**a**) AC impedance measurements of the Fe-HCF, HE-HCFs, and Mn-HCF. Inserted in (**a**) is the equivalent circuit diagram. (**b**) The expanded high-frequency zone is depicted in (**a**). (**c**) CV curves of the Fe_0.60_Mn_0.10_-HCF at a series of scan rates and (**d**) Calculated b values based on CV curves in (**c**). (**e**) GITT curves of HE-HCFs and (**f**) Na^+^ diffusion coefficient of HE-HCFs calculated based on GITT curves in (**e**).

Rate capability is a crucial parameter employed in the assessment of the electrochemical performance of rechargeable battery electrode materials. As shown in Figure 5a, with the specific current increase from 0.05 to 2.0 A·g^−1^, the specific capacities of Fe-HCF, Fe_0.60_Mn_0.10_-HCF, Fe_0.50_Mn_0.20_-HCF, Fe_0.35_Mn_0.35_-HCF, Fe_0.20_Mn_0.50_-HCF, Fe_0.10_Mn_0.60_-HCF, and Mn-HCF decay from 105.6, 114.7, 115.8, 116.5, 108.7, 102.3, and 87.2 mAh·g^−1^ to 51.1, 70.4, 60.0, 55.5, 39.7, 10.2, and 0.1 mAh·g^−1^, which are 48.4%, 61.4%, 52.8%, 47.6%, 34.0%, 10.1%, and 0.1% capacity retentions. The Fe_0.60_Mn_0.10_-HCF has the best rate capability. To sum up, the Fe_0.60_Mn_0.10_-HCF displays the optimal sodium-ion storage performance in considering the ICE, specific capacity, rate capability, and cycling stability due to the synergistic effect of high-entropy and component stoichiometry tuning strategies. The rate performance and cycle stability of the prepared HE-HCFs were compared with those of other reported PBA SIB cathode materials presented in Table S5 [8,34,43,47,48,49,50,51,52]. The Fe_0.60_Mn_0.10_-HCF shows superior rate performance and cycle stability than other PBAs. Figure 5b shows the linear fitting Randles–Sevcik curves of HE-HCFs by plotting the specific peak current in CVs in Figure 6c and Figure S6 as a function of the square root of the sweep rate to determine the apparent sodium-ion diffusion coefficient. The calculated apparent sodium-ion diffusion coefficients of de-sodiation and re-sodiation of HE-HCFs are listed in Table S6, in which the Fe_0.60_Mn_0.10_-HCF shows the highest values of 5.70 × 10^−12^ cm^2^·s^−1^ and 5.57 × 10^−12^ cm^2^·s^−1^, respectively, indicating the fastest kinetics in electrochemical reactions among all the HE-HCFs [53]. The Fe_0.60_Mn_0.10_-HCF, therefore, has the best rate capability. 

### 2.3. Exploring the Origin of the Sodium-Ion Storage Performance Improvement of the HE-HCFs

Figure 6a illustrates the electrochemical impedance spectroscopy (EIS) measurements on Fe-HCF, Fe_0.60_Mn_0.10_-HCF, Fe_0.50_Mn_0.20_-HCF, Fe_0.35_Mn_0.35_-HCF, Fe_0.20_Mn_0.50_-HCF, Fe_0.10_Mn_0.60_-HCF, and Mn-HCF Na half-cells. Their corresponding cell resistances (Rs) are 1.98, 2.08, 2.89, 2.84, 2.68, 3.62, and 2.16 Ω, respectively. The Nyquist curves of the seven materials exhibit comparable trends, comprising a semicircle in the high-frequency region and an inclined straight line in the low-frequency region (Figure 6b). The semicircle represents the charge transfer resistance (Rct), which is related to the electrode material/electrolyte interface reaction. The cell Rct for them are 741, 655, 658, 709, 747, 849, and 1142 Ω, respectively. HE-HCFs have lower Rct but better electrical conductivity than the Fe-HCF and Mn-HCF, and Fe_0.60_Mn_0.10_-HCF has the lowest Rct but the highest electrical conductivity among all the HE-HCFs. Good electrical conductivity leads to enhanced electrochemical performance, especially in terms of kinetics. Therefore, the Fe0.60Mn0.10-HCF has the best rate capability.

A systematic investigation of the electrochemical reaction kinetics of Fe_0.60_Mn_0.10_-HCF as a cathode material for Na half-cells was conducted by testing the corresponding CV curves at varying scan rates (0.2 to 1.6 mV·s^−^¹, Figure 6c). The CV curves of the Fe_0.60_Mn_0.10_-HCF electrode at varying scanning rates display comparable shapes, suggesting that the material exhibits minimal polarization and favorable electrochemical reaction kinetics. By employing the aforementioned formulas (Equations (1) and (2)) [54], the capacitance or diffusion behavior of the electrode can be deduced under different scanning rates (*v*):

*i* = a*v*^b^, or (1)

log *i* = b log *v* + log a (2)

In this context, the constant a represents a constant value, while the value of b represents the capacitance ratio. The latter can be determined by calculating the slope of the logarithmic curve, which is defined as log (*i*) vs. log (*v*). A value of b equal to 0.5 indicates that the process is diffusion-limited, whereas a value of b equal to 1.0 signifies that the process is surface-limited. As presented in Figure 6d, the b values of redox peaks are 0.84 and 0.86, indicating that both Na^+^ insertion and extraction of Fe_0.60_Mn_0.10_-HCF electrode are mixed-controlled processes. CV curves of the remaining four HE-HCFs recorded by increasing the scan rate from 0.2 to 1.6 mV·s^−1^ are shown in Figure S6, and their corresponding b values of redox peaks are shown in Figure S7, which are 0.76 and 0.82 (Fe_0.50_Mn_0.20_-HCF), 0.72 and 0.81 (Fe_0.35_Mn_0.35_-HCF), 0.69 and 0.71 (Fe_0.20_Mn_0.50_-HCF), 0.66 and 0.66 (Fe_0.10_Mn_0.60_-HCF).

To further analyze the Na^+^ diffusion kinetics, galvanostatic intermittent titration technique (GITT) tests were performed on HE-HCFs [55,56], and the results are presented in Figure 6e. During the process of discharging, the Na^+^ diffusion coefficient (DNa^+^) GITT test curves of the five samples exhibited similar trends, and the calculation results are presented in Figure 6f. Obviously, the average Na^+^ diffusion coefficient value of Fe_0.60_Mn_0.10_-HCF (2.58 × 10^−10^ cm^2^·s^−1^) is significantly larger than that of Fe_0.50_Mn_0.20_-HCF (1.98 × 10^−10^ cm^2^·s^−1^), Fe_0.35_Mn_0.35_-HCF (1.81 × 10^−10^ cm^2^·s^−1^), Fe_0.20_Mn_0.50_-HCF (1.56 × 10^−10^ cm^2^·s^−1^), and Fe_0.10_Mn_0.60_-HCF (1.06 × 10^−10^ cm^2^·s^−1^), which significantly boosts the rate capability of the Fe_0.60_Mn_0.10_-HCF electrode during the sodiation/de-sodiation [57]. Interestingly, the apparent sodium-ion diffusion coefficients of de-sodiation and re-sodiation of the Fe_0.60_Mn_0.10_-HCF are 5.70 × 10^−12^ cm^2^·s^−1^, and 5.57 × 10^−12^ cm^2^·s^−1^, which are more than two orders of magnitude higher than those obtained via GITT (2.58 × 10^−10^ cm^2^·s^−1^). Disparities between values acquired using CV and GITT are not uncommon [58] and reflect the different conditions under which they are obtained: the Randles–Sevcik method subjects the material to continuous and much higher voltage differentials while GITT is performed at low currents and near equilibrium conditions [59]. 

## 3. Materials and Methods

### Synthesis of Metal Hexacyanoferrate

Single-metal PBs (Fe-HCF and Mn-HCF) and HE-HCFs were synthesized using the sodium citrate-chelating agent co-precipitation method. Taking the Na_1.156_Fe_0.599_Mn_0.095_Ni_0.092_Cu_0.109_Zn_0.105_ [Fe(CN)_6_]_0.724_·3.11H_2_O (Fe_0.60_Mn_0.10_-HCF) as an example, firstly, NaCl (15 g) and Na_4_Fe(CN)_6_ (1.823 g, 6 mmol) were accurately weighed and dissolved in 100 mL of deionized water and then stirred evenly to form solution A. Secondly, C_6_H_5_Na_3_O_7_·2H_2_O (1.470 g, 5 mmol) and ascorbic acid (0.5 g) were weighed and dissolved in 100 mL of deionized water and then FeCl_2_·4H_2_O (0.497 g, 3.0 mmol), MnCl_2_·4H_2_O (0.098 g, 0.5 mmol), NiCl_2_·6H_2_O (0.119 g, 0.5 mmol), CuCl_2_·2H_2_O (0.085 g, 0.5 mmol), and ZnCl_2_ (0.068 g, 0.5 mmol) were added and stirred evenly to form solution B. Solution B was added in its entirety to Solution A, which was continuously stirred throughout the process. The combined solution was agitated for 2 h. The obtained suspension was then aged for 12 h at room temperature and was then centrifugated to obtain precipitate, which was then washed with deionized water and anhydrous ethanol alternatively several times and was dried in a vacuum drying oven at 120 °C for 12 h. Single-metal PBs (Fe-HCF and Mn-HCF) and other HE-HCFs with different component stoichiometry of Fe and Mn (Fe_0.60_Mn_0.10_-HCF, Fe_0.50_Mn_0.20_-HCF, Fe_0.35_Mn_0.35_-HCF, Fe_0.20_Mn_0.50_-HCF, and Fe_0.10_Mn_0.60_-HCF) electrode materials were prepared using the same synthesis method, with alterations made to the type and mass ratio of the added metal salts. All reagents mentioned above were of analytical grade and used without further purification, all of which were capurchased from commercial sources (Sinopharm Chemical Reagent Co., Ltd., Shanghai, China).

Comprehensive details regarding material characterization, electrochemical measurements, and kinetics analysis can be found in the Supplementary Materials.

## 4. Conclusions

In this study, the single-metal Fe-HCF and Mn-HCF and HE-HCFs containing Fe, Mn, Ni, Cu, and Zn were synthesized using a facile and economic co-precipitation method at room temperature. They are used as cathode materials for SIBs. Battery performance test results show that the HE-HCFs show significantly improved sodium-ion storage performance than the single-metal Fe-HCF and Mn-HCF due to the high-entropy and component stoichiometry tuning strategies, which reduce the crystalline water content and improve the electrical conductivity and kinetics of materials demonstrated using the EIS and GITT analysis. The component and stoichiometry optimized HE-HCF of Fe_0.60_Mn_0.10_-HCF, therefore, displays the most outstanding ICE, specific capacity, rate capability, and cycling stability. Our findings could guide the design and development of low-cost, Co-free, high-entropy SIB cathode materials with high performance.

## Figures and Tables

**Table 1:** Chemical formulas and corresponding abbreviated forms of all the synthesized Fe-HCF, Mn-HCF, and HE-HCFs.

Sample	Chemical Formula
Fe-HCF	Na_1.247_Fe [Fe(CN)_6_]_0.791_·3.61H_2_O
Fe_0.60_Mn_0.10_-HCF	Na_1.156_Fe_0.599_Mn_0.095_Ni_0.092_Cu_0.109_Zn_0.105_ [Fe(CN)_6_]_0.724_·3.11H_2_O
Fe_0.50_Mn_0.20_-HCF	Na_1.142_Fe_0.505_Mn_0.185_Ni_0.094_Cu_0.111_Zn_0.105_ [Fe(CN)_6_]_0.728_·3.09H_2_O
Fe_0.35_Mn_0.35_-HCF	Na_1.152_Fe_0.373_Mn_0.314_Ni_0.092_Cu_0.116_Zn_0.104_ [Fe(CN)_6_]_0.744_·3.14H_2_O
Fe_0.20_Mn_0.50_-HCF	Na_1.157_Fe_0.234_Mn_0.460_Ni_0.091_Cu_0.109_Zn_0.106_ [Fe(CN)_6_]_0.759_·3.45H_2_O
Fe_0.10_Mn_0.60_-HCF	Na_1.110_Fe_0.138_Mn_0.550_Ni_0.102_Cu_0.109_Zn_0.101_ [Fe(CN)_6_]_0.741_·3.37H_2_O
Mn-HCF	Na_0.706_Mn [Fe(CN)_6_]_0.857_·2.83H_2_O

## Data Availability

The data presented in this study are available upon request from the corresponding author.

## Supplementary Materials

The following supporting information can be downloaded at: https://www.mdpi.com/article/10.3390/molecules29194559/s1, Figure S1: TGA curves recorded for Fe-HCF, HE-HCFs, and Mn-HCF under Ar flow; Figure S2: The XRD corresponds to the Rietveld refinement profile of Fe_0.50_Mn_0.20_-HCF material; Figure S3: SEM images of Fe-HCF, HE-HCFs, and Mn-HCF; Figure S4: XPS survey spectrum and C1s spectra of fresh Fe_0.60_Mn_0.10_-HCF powder; Figure S5: The change of average working voltage of HE-HCFs material during discharge process. Figure S6: CVs at a series of scan rates of Fe_0.50_Mn_0.20_-HCF, Fe_0.35_Mn_0.35_-HCF, Fe_0.20_Mn_0.50_-HCF, and Fe_0.10_Mn_0.60_-HCF electrode; Figure S7: Calculated b values of Fe_0.50_Mn_0.20_-HCF, Fe_0.35_Mn_0.35_-HCF, Fe_0.20_Mn_0.50_-HCF, and Fe_0.10_Mn_0.60_-HCF electrode. Table S1: ICP-OES of Fe-HCF, HE-HCFs, and Mn-HCF; Table S2: EA of CHN analysis for Fe-HCF, HE-HCFs, and Mn-HCF; Table S3: The refined structure of Fe_0.60_Mn_0.10_-HCF; Table S4: The refined structure of Fe_0.50_Mn_0.20_-HCF; Table S5: Representative performance of reported PBAs cathodes for SIBs; Table S6: Calculated peaks diffusion coefficient (cm^2^·s^−1^) of HE-HCFs based on CV curves.
