# Supplementary material for: High-Entropy and Component Stoichiometry Tuning Strategies Boost the Sodium-Ion Storage Performance of Cobalt-Free Prussian Blue Analogues Cathode Materials
Source: Molecules. 2024 Sep 25;29(19):4559. doi: 10.3390/molecules29194559 (PMC11478298; doi:10.3390/molecules29194559)
Supplement: Supplementary file 1 [file molecules-29-04559-s001.zip › molecules-3207635-supplementary.pdf]

Supplementary Materials

High-Entropy and Component Stoichiometry Tuning Strategies Boost the Sodium-Ion Storage Performance of Cobalt-free Prussian Blue Analogues Cathode Materials

Yuan-Ting Lin [1], Bai-Tong Niu [1,2], Zi-Han Wang [3], Yu-Xi Li [2], Yun-Peng Xu [4], Shi-Wei Liu [1], Yan-Xin Chen [5] and Xiu-Mei Lin [1,*]

1 College of Chemistry, Chemical Engineering and Environment, Minnan Normal University, Zhangzhou 363000, China

2 State Key Laboratory of Physical Chemistry of Solid Surfaces, College of Chemistry and Chemical Engineering, iChEM, Xiamen University, Xiamen 361005, China

3 College of Energy, Xiamen University, Xiamen 361005, China

4 Department of Physic, Xiamen University, Xiamen 361005, China

5 State Key Laboratory of Structural Chemistry, Fujian Institute of Research on the Structure of Matter, Chinese Academy of Sciences, Fuzhou 350002, China

* Correspondence: xiu-mei.lin@xmu.edu.cn

Experimental Section

Material Characterization

XRD patterns, FTIR spectra, and Raman spectra of the PBA materials were recorded using a Rigaku D/MAX-RB X-ray diffractometer with Cu Kα radiation, a NICOLET iS 10IR Fourier transform infrared spectrometer, and a HORIBA XploRA PLUS Raman spectrometer, respectively. Morphological features of the PBA materials were examined using a Regulus 8100 field-emission scanning electron microscopy (SEM) and a Tecnai F30 high-resolution transmission electron microscopy (HRTEM). The thermal stability of PBA materials was evaluated using a thermogravimetric analysis (Mettler TGA/DSC 3+) at a rate of 5 K • min^{-1} in argon. To confirm the formula of PBAs, ICP-OES (Agilent 730) was used to measure the concentration of Na, Fe, Mn, Ni, Cu, and Zn. Elemental analysis (EA, Elementar UNICUBE) was used to analyze the content of C, H, and N. The valence evolution of the transition metal was revealed by the X-ray photoelectron spectra (XPS, ESCALAB 250Xi).

The formulas of PBAs are calculated according to the following formula based on the obtained TGA, ICP-OES, and EA data:

$$n_{(\mathrm{CN})_6} = \frac{Nwt\%}{M_N * 6}$$

$$\mathrm{Metal\%} = \mathrm{Na\%} + \mathrm{Fe\%} + \mathrm{Mn\%} + \mathrm{Co\%} + \mathrm{Ni\%} + \mathrm{Cu\%}$$

$$n_{TM_{HS}} = \frac{TM\% / Metal\% \quad (1 - Cwt\% - Nwt\% - 9 * Hwt\%)}{M_{TM}}$$

$$n_{\mathrm{Fe}_{\mathrm{total}}} = \frac{Fe\% / Metal\% \quad (1 - Cwt\% - Nwt\% - 9 * Hwt\%)}{M_{Fe}}$$

$$n_{\mathrm{Na}} = \frac{Na\% / Metal\% \quad (1 - Cwt\% - Nwt\% - 9 * Hwt\%)}{M_{Na}}$$

$$n_{\mathrm{Fe}_{HS}} = n_{\mathrm{Fe}_{total}} - n_{(CN)_6}$$

$$n_{H_2O} = \frac{Hwt\%}{2 * M_H}$$

$n_{\mathrm{Fe}_{HS}}$ Stands for the high-spin Fe.

All the calculated results are normalized by $\sum TM_{HS}$.

Electrochemical Characterization

The cathode was prepared by mixing and stirring 70 wt% synthesized PBAs, 20 wt% Super P, and 10 wt% polyvinylidene fluoride (PVDF) with N-methyl pyrrolidinone (NMP) mechanically to form a homogeneous slurry and was then coated onto an Al foil. After being dried at 120 °C for 24 h in a vacuum oven, a load of the electrode of about 1.25 mg·cm^{-2} was obtained. The 2032 stainless steel coin cells were assembled in an Argon-filled glove box (Vigor) with O_2 and H_2O levels maintained below 0.1 ppm. Sodium half-cells were assembled using PBAs cathode, glass fiber (Whatman, GF/C), sodium foil, and 1.0 M $NaClO_4$ in EC: PC=1: 1 Vol% with 5%FEC as the working electrode, separator, counter electrode, and electrolyte, respectively. Cyclic voltammetry (CV) and AC electrochemical impedance spectroscopy (EIS) measurements were conducted using an Autolab electrochemical workstation. Galvanostatic charge-discharge test at different specific currents and galvanostatic intermittent titration technique (GITT) test were performed by the Neware battery testing system with a voltage range of 2.0 to 4.0 V at 25°C. Before testing, fresh cells were first cycled under a constant specific current of 10 mA·g^{-1} for 5 cycles to reach the normal state. Afterward, the single GITT test was conducted at a steady current pulse of 10 mA·g^{-1}. In the charging process, the battery was charged for 20 min, then interrupted for 40 min. The discharge process was carried out according to the same procedure.

Calculation of the sodium-ion diffusion coefficient (*D*) by Randles-Sevcik equation:

The sodium-ion diffusion coefficient (*D*) was calculated by analyzing the peak current (I_p) from cyclic voltammetry measurements (Figure 6c and Figure S5) using the Randles-Sevcik equation [53]:

$$I_{\mathrm{p}} = 2.69\times 10^{5} n^{3/2} C A D^{1/2} v^{1/2}$$

where n represents the number of electrons per redox reaction; *C* is the Na^+ molar concentration in the cathode material (mol·cm^{-3}); *A* refers to the electrode surface area (cm^{-2}); *D* is the apparent diffusion coefficient (cm^2·s^{-1}), and v is the specific scanning rate (mV·s^{-1}).

Calculation of the sodium-ion diffusion coefficient (*D*) by galvanostatic intermittent titration technique (GITT) tests (Figure 6e)

The Na^+ diffusion coefficient is calculated by the following formula [55,56]:

$$D = \frac{4}{\pi\tau}\left(\frac{m_B V_M}{M_B S}\right)^2\left(\frac{\Delta E_S}{\Delta E_\tau}\right)^2 \quad \left(\tau << L^2 / D_{Na^+}\right)$$

where τ, m_B, V_M, M_B, *S*, and *L* denote the current pulse time (s), the mass of the active material (g), molar volume (cm^3·mol^{-1}), relative molecular mass (g·mol^{-1}), electrode–electrolyte contact area (cm^2), and electrode thickness, respectively. ΔE_S and ΔE_τ are the voltage change caused by the pulse and the voltage change during the constant current charge/discharge (V), respectively.

Supplementary Figures

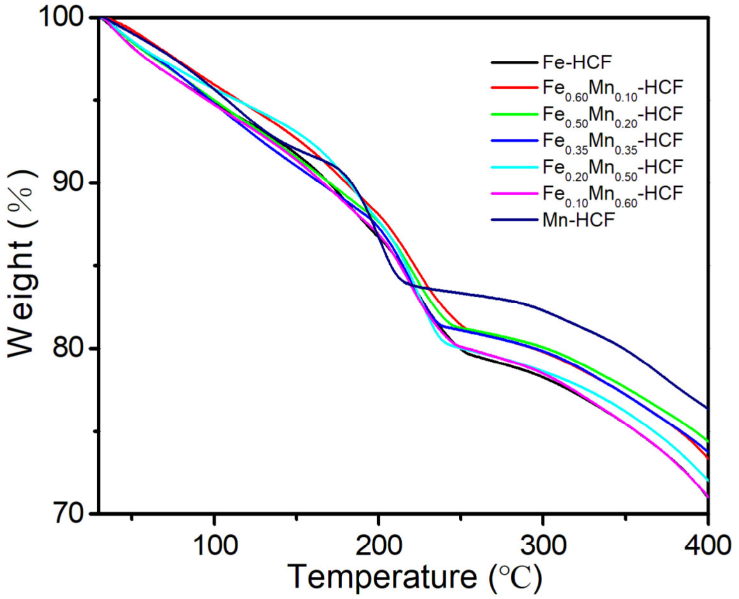

Figure S1. TGA curves were recorded for Fe-HCF, HE-HCFs, and Mn-HCF under Ar flow.

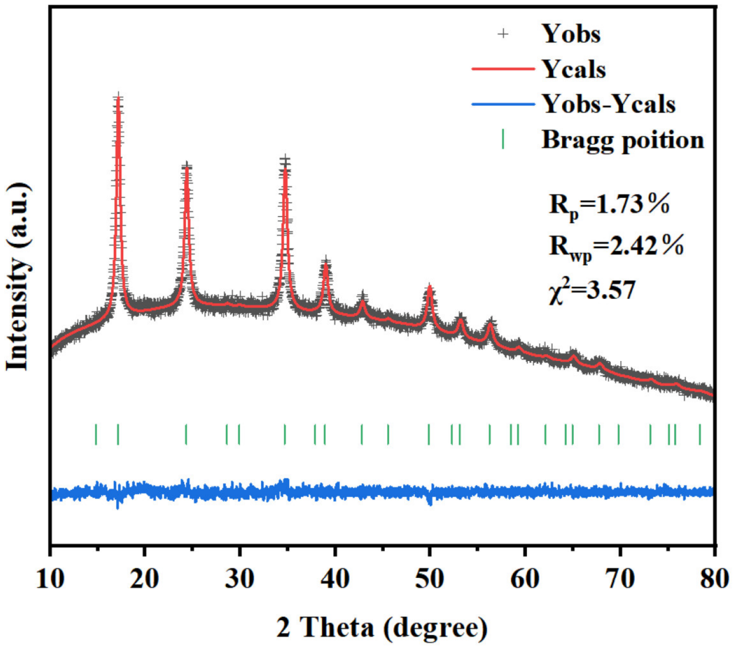

Figure S2. The XRD corresponds to the Rietveld refinement profile of $Fe_{0.50}Mn_{0.20}$-HCF material.

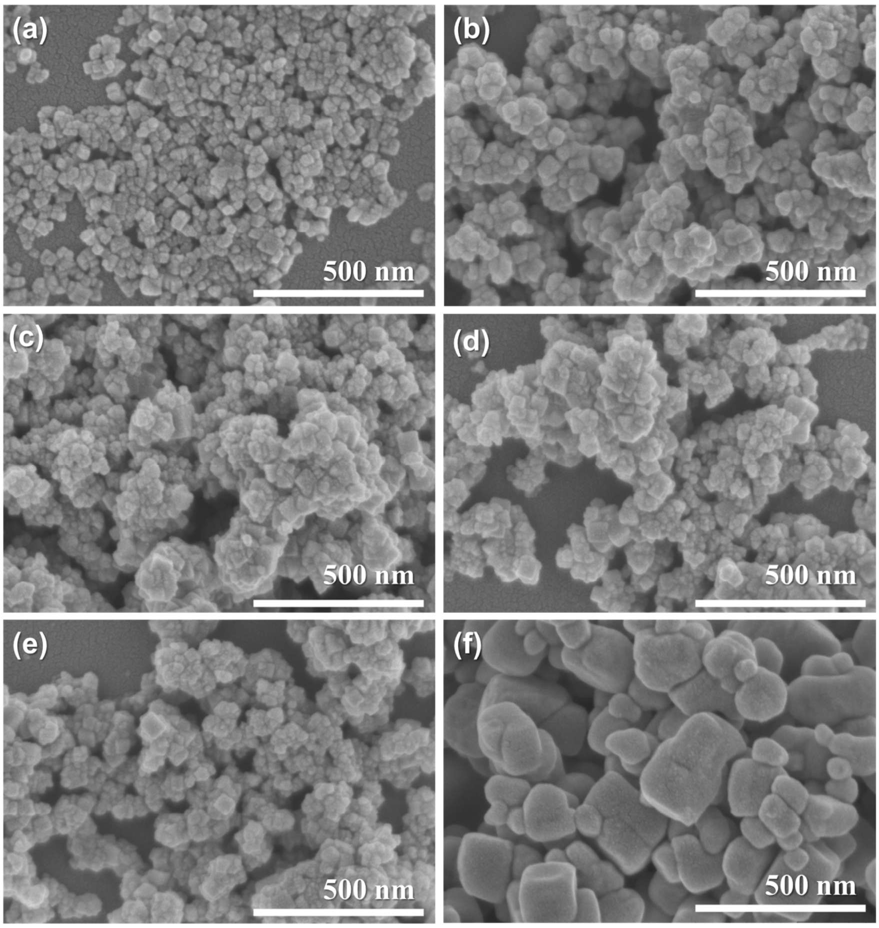

Figure S3. SEM images of (a) Fe-HCF, (b) $Fe_{0.50}Mn_{0.20}$-HCF, (c) $Fe_{0.35}Mn_{0.35}$-HCF, (d) $Fe_{0.20}Mn_{0.50}$-HCF, (e) $Fe_{0.10}Mn_{0.60}$-HCF, and (f) Mn-HCF.

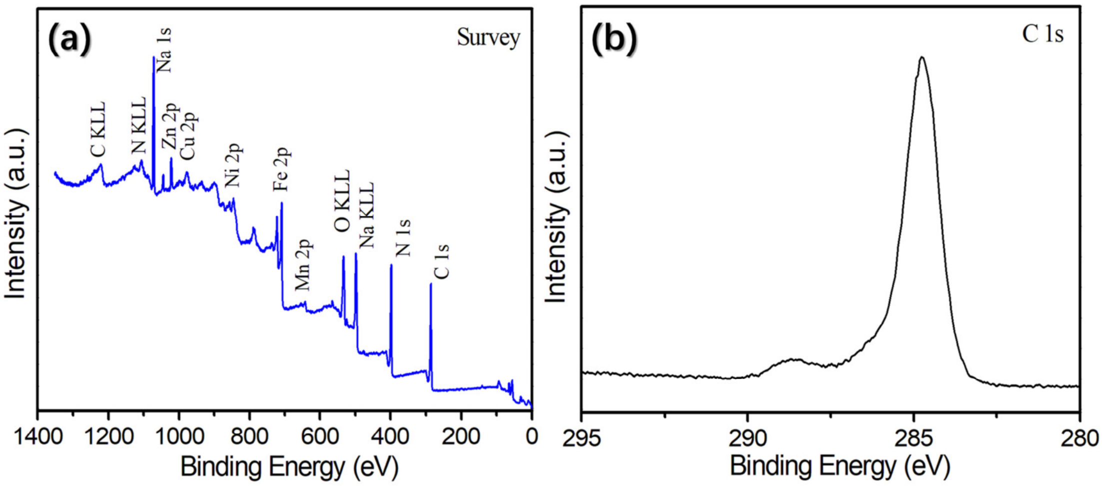

Figure S4. (a) XPS survey spectrum and (b) C1s spectra of fresh $Fe_{0.60}Mn_{0.10}$-HCF powder.

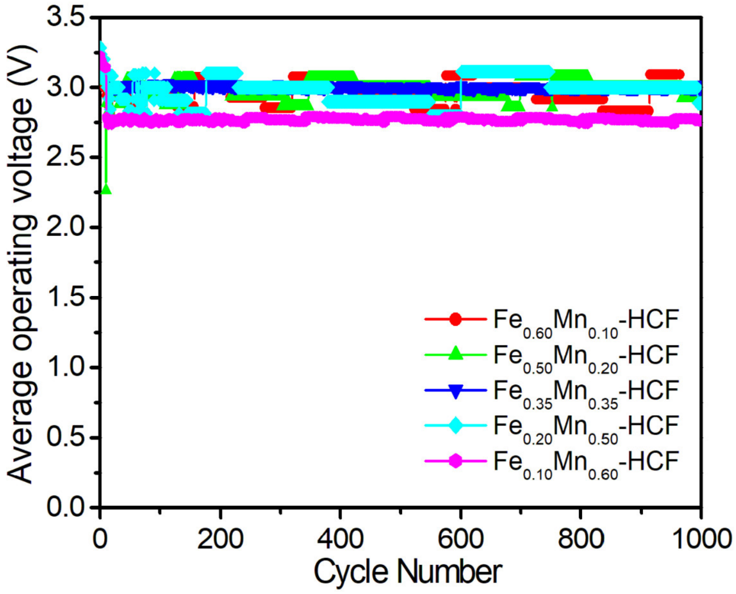

Figure S5. The change of the average working voltage of HE-HCFs materials during the electrochemical cycling process.

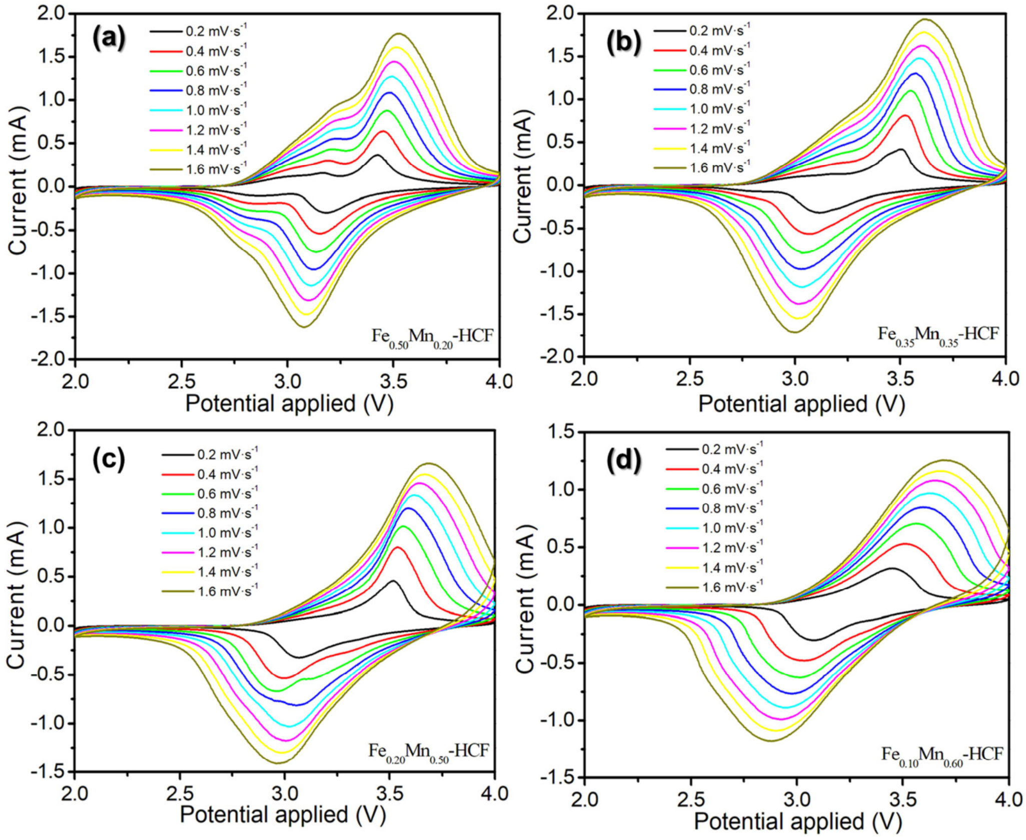

Figure S6. CVs at a series of scan rates of (a) $Fe_{0.50}Mn_{0.20}$-HCF, (b) $Fe_{0.35}Mn_{0.35}$-HCF, (c) $Fe_{0.20}Mn_{0.50}$-HCF, and (d) $Fe_{0.10}Mn_{0.60}$-HCF electrode.

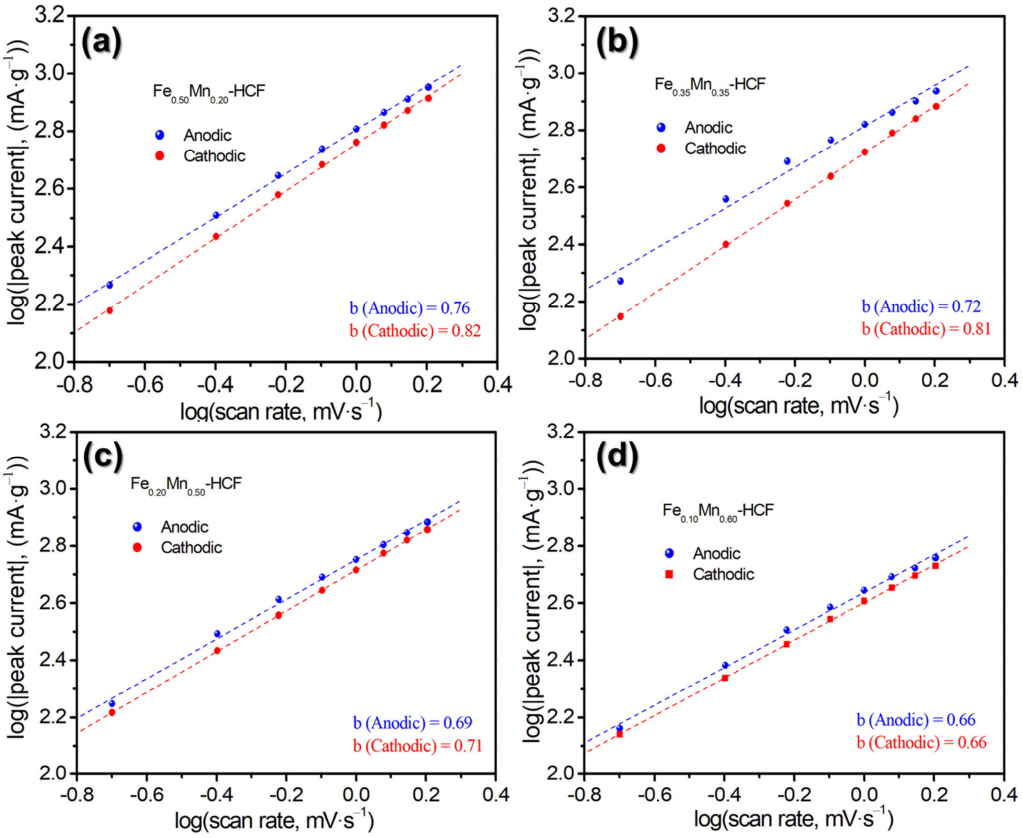

Figure S7. Calculated b value of (a) $Fe_{0.50}Mn_{0.20}$-HCF, (b) $Fe_{0.35}Mn_{0.35}$-HCF, (c) $Fe_{0.20}Mn_{0.50}$-HCF, and (d) $Fe_{0.10}Mn_{0.60}$-HCF electrode.

Table S1. ICP-OES of Fe-HCF, HE-HCFs, and Mn-HCF.

Sample	Na (wt%)	Fe (wt%)	Mn (wt%)	Ni (wt%)	Cu (wt%)	Zn (wt%)
Fe-HCF	7.37	25.72	—	—	—	—
$Fe_{0.60}Mn_{0.10}$-HCF	7.98	22.20	1.56	1.62	2.08	2.07
$Fe_{0.50}Mn_{0.20}$-HCF	8.02	21.04	3.10	1.69	2.15	2.10
$Fe_{0.35}Mn_{0.35}$-HCF	8.04	18.93	5.24	1.64	2.24	2.07
$Fe_{0.20}Mn_{0.50}$-HCF	7.66	15.98	7.27	1.54	2.00	1.99
$Fe_{0.10}Mn_{0.60}$-HCF	7.49	14.41	8.86	1.75	2.04	1.94
Mn-HCF	7.59	13.47	14.82	—	—	—

Table S2. EA of CHN analysis for Fe-HCF, HE-HCFs, and Mn-HCF.

Sample	C (wt%)	N (wt%)	H (wt%)
Fe-HCF	21.47	21.23	1.802
$Fe_{0.60}Mn_{0.10}$-HCF	20.24	20.99	1.746
$Fe_{0.50}Mn_{0.20}$-HCF	20.12	21.06	1.767
$Fe_{0.35}Mn_{0.35}$-HCF	20.22	21.19	1.774
$Fe_{0.20}Mn_{0.50}$-HCF	20.34	21.28	1.798
$Fe_{0.10}Mn_{0.60}$-HCF	20.21	21.06	1.854
Mn-HCF	20.99	21.60	1.949

Table S3. The refined structure of $Fe_{0.60}Mn_{0.10}$-HCF.

$Fe_{0.60}Mn_{0.10}$-HCF ($Na_{1.156}Fe_{0.599}Mn_{0.095}Ni_{0.092}Cu_{0.109}Zn_{0.105}[Fe(CN)_6]_{0.724}$·$3.11H_2O$).

Cubic. S.G. Fm-3m a=b=c=10.3190(7) Å, α=β=γ=90.0° (R_w = 2.01%, R_{wp} = 1.59%)

Atom	X	Y	Z	Mult	Occ.	ADP (U_{iso} / Å^2)
Mn	0.50000	0.00000	0.00000	2	0.095	0.0100(19)
Ni	0.50000	0.00000	0.00000	2	0.092	0.0100(19)
Cu	0.50000	0.00000	0.00000	2	0.109	0.0100(19)
Zn	0.50000	0.00000	0.00000	2	0.105	0.0100(19)
Fe1	0.00000	0.00000	0.00000	2	0.667(8)	0.0144
Fe2	0.50000	0.00000	0.00000	2	0.599	0.0285(19)
C1	0.19195(22)	0.00000	0.00000	4	0.667(8)	0.0540
N1	0.30685(34)	0.00000	0.00000	4	0.667(8)	0.0540
Na	0.25000	0.25000	0.25000	4	1	0.1020(41)

Table S4. The refined structure of $Fe_{0.50}Mn_{0.20}$-HCF.

$Fe_{0.50}Mn_{0.20}$-HCF ($Na_{1.142}Fe_{0.505}Mn_{0.185}Ni_{0.094}Cu_{0.111}Zn_{0.105}[Fe(CN)_6]_{0.728}\cdot 2.55H_2O$). Cubic. S.G. Fm-3m a=b=c=10.3501(6) Å, α=β=γ=90.0° (R_p = 1.73%, R_{wp} = 2.42%)						
**Atom**	**X**	**Y**	**Z**	**Mult**	**Occ.**	**ADP (U_{iso} / Å^2)**
Mn	0.50000	0.00000	0.00000	2	0.185	0.0100(19)
Ni	0.50000	0.00000	0.00000	2	0.094	0.0100(19)
Cu	0.50000	0.00000	0.00000	2	0.111	0.0100(19)
Zn	0.50000	0.00000	0.00000	2	0.105	0.0100(19)
Fe1	0.00000	0.00000	0.00000	2	0.667(8)	0.0144
Fe2	0.50000	0.00000	0.00000	2	0.505	0.0285(19)
C1	0.19353(21)	0.00000	0.00000	4	0.667(8)	0.0540
N1	0.30805(32)	0.00000	0.00000	4	0.667(8)	0.0540
Na	0.25000	0.25000	0.25000	4	1	0.1020(41)

Table S5. Representative performance of reported PBAs cathodes for SIBs.

Material	Rate capability ($mAh·g^{-1}$@ $A·g^{-1}$)	Cycle Life (Retention, Cycles, Rate)	Ref.
Fe-HCF	105.6/51.1 @ 0.05/2.0	47.4%, 1000th, 1.0 $A·g^{-1}$	This Work
$Fe_{0.60}Mn_{0.10}$-HCF	114.6/70.4 @ 0.05/2.0	66.7%, 1000th, 1.0 $A·g^{-1}$	
$Fe_{0.50}Mn_{0.20}$-HCF	115.0/60.0 @ 0.05/2.0	72.5%, 1000th, 1.0 $A·g^{-1}$	
$Fe_{0.35}Mn_{0.35}$-HCF	116.5/55.5 @ 0.05/2.0	70.3%, 1000th, 1.0 $A·g^{-1}$	
$Fe_{0.20}Mn_{0.50}$-HCF	108.7/39.7 @ 0.05/2.0	70.6%, 1000th, 1.0 $A·g^{-1}$	
$Fe_{0.10}Mn_{0.60}$-HCF	102.3/10.2 @ 0.05/2.0	58.8%, 1000th, 1.0 $A·g^{-1}$	
Mn-HCF	87.2/0.1 @ 0.05/2.0	36.8%, 1000th, 1.0 $A·g^{-1}$	
HQ-PB	90.50/63.89@ 0.01/2.0	89%, 300th, 0.05 $A·g^{-1}$	[47]
$Na_{1.38}Mn[Fe(CN)_6]_{0.92}□_{0.08}·2.57H_2O$	123.8/73.8 @ 0.025/0.6	70%, 500th, 0.2 $A·g^{-1}$	[34]
$Na_{1.93}Mn[Fe(CN)_6]·2.38H_2O$	156.16/53.92 @ 0.01/0.5	49.35%, 500th, 0.1 $A·g^{-1}$	[48]
$Na_{1.02}Mn_{0.57}Fe_{0.43}[Fe(CN)_6]_{0.63}·1.39H_2O$	127/70 @ 0.025/1.2	86%, 200th, 0.1 $A·g^{-1}$	[49]
$Na_{1.52}Mn_{0.94}Zn_{0.06}[Fe(CN)_6]_{0.91}·2.01H_2O$	126/~58.5 @ 0.05/1.6	70%, 1000th, 0.8 $A·g^{-1}$	[43]
$Na_{1.71}Ni_{0.07}Co_{0.93}[Fe(CN)_6]_{0.94}□_{0.06}·1.92H_2O$	146/105 @ 0.02/1.0	85%, 100th, 0.1 $A·g^{-1}$	[50]
SC–HE-PBA ($Na_{1.70}Fe_{0.2}Mn_{0.2}Co_{0.2}Ni_{0.2}Cu_{0.2}[Fe(CN)_6]_{0.98}□_{0.02}·2.35H_2O$)	96.5/74.4 @ 1.0/3.0	76.1%, 500th, 0.5 $A·g^{-1}$	[25]

$Na_{1.83}Cu_{0.12}Mn_{0.88}[Fe(CN)_6]_{1.00}·0.48H_2O$	120/99.6 @ 0.1 C/10 C (1C = 170 mA·g^{-1})	67.6%, 500th, 10 C (1C = 170 mA·g^{-1})	[51]
Ni-V$_{[Fe(CN)6]}$-Mn-PBA	98/18 @ 0.5 C/2 C (1C = 100 mA·g^{-1})	71%, 100th, 0.5 C (1C = 100 mA·g^{-1})	[52]

Table S6. Calculated peaks diffusion coefficient (cm^2·s^{-1}) of HE-HCFs based on CV curves.

**Sample**	**De-sodiation**	**Re-sodiation**
$Fe_{0.60}Mn_{0.10}$-HCF	5.70×10^{-12}	5.57×10^{-12}
$Fe_{0.50}Mn_{0.20}$-HCF	4.48×10^{-12}	4.03×10^{-12}
$Fe_{0.35}Mn_{0.35}$-HCF	3.87×10^{-12}	3.50×10^{-12}
$Fe_{0.20}Mn_{0.50}$-HCF	2.99×10^{-12}	2.78×10^{-12}
$Fe_{0.10}Mn_{0.60}$-HCF	1.62×10^{-12}	1.44×10^{-12}

References

25. Huang, Y.; Zhang, X.; Ji, L.; Wang, L.; Xu, B.-B.; Shahzad, M.-W.; Tang, Y.-X.; Zhu, Y.-F.; Yan, M.; Sun, G.-X.; et al. Boosting the sodium storage performance of prussian blue analogs by single-crystal and high-entropy approach. *Energy Storage Mater.* **2023**, *58*, 1–8.
34. Tang, Y.; Li, W.; Feng, P.-Y.; Zhou, M.; Wang, K.-L.; Wang, Y.-S.; Zaghib, K.; Jiang, K. High-performance manganese hexacyanoferrate with cubic structure as superior cathode material for sodium-ion batteries. *Adv. Funct. Mater.* **2020**, *30*, 1908754.
43. Wang, M.-L.; Ling, R.; Yang, C.; Qi, W.-T. Structural regulation of Mn-based prussian blue induced by zinc-substitution for enhanced sodium storage performance. *Electrochim. Acta* **2023**, *462*, 142711.
47. Li, Q.-Y.; Xu, C.-M.; Liang, Y.-R.; Yang, Z.; LeGe, N.; Peng, J.; Chen, L.-J.; Lai, W.-H.; Wang, Y.-X.; Tao, Z.-L.; et al. Reforming magnet waste to Prussian blue for sustainable sodium-ion batteries. *ACS Appl. Mater. Interfaces* **2022**, *14*, 47747–47757.
48. Lin, K.; He, Z.-M.; Shen, L.; Su, J.-B.; Huang, Z.-Y.; Xia, Y.-M.; Wang, Y. Preparation high-performance cathode of vacancy-free Prussian blue analogues for sodium ion batteries by directional capture of free Mn-ion. *J. Electroanal. Chem.* **2024**, *966*, 118395.
49. Wang, J.; Wang, Z.-P.; Liu, H.; Gao, J.-F.; Xu, Y.-T.; Chen, Z.; Li, X.-L.; Liu, Y. Synthesis of Fe-doped Mn-based Prussian blue hierarchical architecture for high-performance sodium ion batteries. *Electrochim. Acta* **2023**, *448*, 142183.

50. Quan, J.-J.; Xu, E.-Z.; Zhu, H.-W.; Chang, Y.-J.; Zhu, Y.; Li, P.-C.; Sun, Z.-J.; Yu, D.-B.; Jiang, Y. A Ni-doping-induced phase transition and electron evolution in cobalt hexacyanoferrate as a stable cathode for sodium-ion batteries. *Phys. Chem. Chem. Phys.* **2021**, *23*, 2491–2499.
51. Lee, J.; Baek, J.; Kim, Y.; Jeong, W.; Kim, H.; Oh, G.; Oh, Y.; Jeong, S.; Kansara, S.; Sambandam, B.; et al. Cu-substituted Prussian white with low crystal defects as high-energy cathode materials for sodium-ion batteries. *Mater. Today Chem.* **2023**, *33*, 101741.
52. El-Hady, D.-A.; Lyu, Y.-X.; Zhan, S.-K.; Yang, J.-X.; Wang, Y.; Yang, F.; Zhao, Q.-L.; Gu, M.; Shao, M.-H. Vacancy and composition engineering of manganese hexacyanoferrate for sodium-ion storage. *ACS Appl. Energy Mater.* **2022**, *5*, 8547–8553.
53.Yang, J.-W.; Yan, B.; Ye, J.; Li, X.; Liu, Y.; You, H.-P. Carbon-coated $LiCrTiO_4$ electrode material promoting phase transition to reduce asymmetric polarization for lithium-ion batteries. *Phys. Chem. Chem. Phys.* **2014**, *16*, 2882–2891.
55. Peng, F.-W.; Yu, L.; Gao, P.-Y.; Liao, X.-Z.; Wen, J.-G.; He, Y.-S.; Tan, G.-Q.; Ren, Y.; Ma, Z.-F. Highly crystalline sodium manganese ferrocyanide microcubes for advanced sodium ion battery cathodes. *J. Mater. Chem. A* **2019**, *7*, 22248–22256.
56. Hou, X.-H.; Huang, Y.-L.; Ma, S.-M.; Zou, X.-L.; Hu, S.-J.; Wu, Y.-P. Facile hydrothermal method synthesis of coralline-like $Li_{1.2}Mn_{0.54}Ni_{0.13}Co_{0.13}O_2$ hierarchical architectures as superior cathode materials for lithium-ion batteries. *Mater. Res. Bull.* **2015**, *63*, 256–264.
